# Protein Kinase B Inactivation Is Associated with Magnolol-Enhanced Therapeutic Efficacy of Sorafenib in Hepatocellular Carcinoma In Vitro and In Vivo

**DOI:** 10.3390/cancers12010087

**Published:** 2019-12-30

**Authors:** Jiann-Hwa Chen, I-Tsang Chiang, Fei-Ting Hsu

**Affiliations:** 1 Department of Emergency Medicine, Cathay General Hospital, Taipei 280, Taiwan; cgh08335@cgh.org.tw; 2 School of Medicine, Fu Jen Catholic University, New Taipei City 242, Taiwan; 3 Department of Radiation Oncology, Show Chwan Memorial Hospital, Changhua 500, Taiwan; 4 Department of Radiation Oncology, Chang Bing Show Chwan Memorial Hospital, Changhua 505, Taiwan; 5 Department of Medical Imaging and Radiological Sciences, Central Taiwan University of Science and Technology, Taichung 406, Taiwan; 6 Department of Biological Science and Technology, China Medical University, Taichung 404, Taiwan

**Keywords:** magnolol, sorafenib, AKT, hepatocellular carcinoma

## Abstract

Although sorafenib, an oral multikinase inhibitor, was approved as a treatment drug of advance hepatocellular carcinoma (HCC), treatment efficacy still requires improvement. Searching for the adjuvant reagent for enhancing sorafenib efficacy remains as a critical issue. Sorafenib has been proved to suppress extracellular signal-regulated kinases (ERK) in HCC; however, protein kinase B (AKT) was not affected by it. Targeting AKT in combination with sorafenib could be an important breakthrough point of HCC treatment. Many herbal compounds and composite formulas have been shown to enhance anti-HCC activity of sorafenib. Magnolol is a bioactive compound extracted from the bark of the *Magnolia officinalis* and has been shown to induce apoptosis and inhibit cell invasion in HCC in vitro. However, whether magnolol sensitizes HCC to sorafenib is ambiguous. In this study, we indicated that magnolol significantly enhanced sorafenib-diminished tumor cell growth, expression of anti-apoptotic proteins, and migration/invasion ability compared to sorafenib alone. Magnolol significantly boosted sorafenib-induced extrinsic/intrinsic dependent apoptosis pathways in HCC. Notably sorafenib could not reduce protein level of AKT (Ser473), but expression of AKT (Ser473) was significantly decreased by magnolol or magnolol combined with sorafenib. LY294002 as specific AKT inhibitor was used to confirm that AKT inactivation may promote anticancer effect of sorafenib. Taken together, AKT inhibition is associated with magnolol-enhanced the therapeutic effect of sorafenib in HCC. We suggested magnolol as the potential adjuvant which may enhance therapeutic benefits of sorafenib in patients with HCC.

## 1. Introduction

Herbal medicines, natural products from medicinal plants, can reduce inflammatory response and be used to treat liver disease [1]. Many herbal compounds and composite formula are chemopreventive agents that prevent liver carcinogenesis in patients with cirrhosis. Both glycyrrhizin (aqueous extract of the liquorice root) and sho saiko-to (TJ-9) have been found to be effective in inhibiting development of hepatocellular carcinoma (HCC) [2,3]. In addition, herbal medicines are also recognized as potential complementary treatments for HCC [4].

Herbal compounds and composite formula inhibit tumor growth through induction of apoptosis, cell cycle arrest and blockage of intracellular signal transduction in HCC [5]. Curcumin, a flavonoid extracted from *Curcuma longa*, has been indicated to induce apoptosis, cell cycle arrest, and reduce angiogenic and metastatic activity leading to the inhibition of tumor progression in HCC in vitro and in vivo [6,7,8]. Sho-saiko-to also diminishes cell proliferation by inducing apoptosis and cell cycle arrest in HCC [9]. Some clinical trials presented herbal medicines can offer therapeutic benefits for patients with HCC. Tsuchiya et al. found Shi-Quan-Da-Bu-Tang (TJ-48) prologs hepatic recurrence-free survival in patients with HCC [10]. Lin et al. presented shenqi mixture (SQM), an herbal composite formula, enhances anti-HCC efficacy of microwave coagulation therapy and improves survival rate and patients’ symptoms such as hepatic region pain, fever, weakness, poor appetite, and jaundice [11].

Sorafenib (Nexavar), the oral multi-kinase inhibitor, has been approved for the treatment of advanced HCC and renal cell carcinoma [12]. Some studies presented herbal medicines can enhance anti-HCC efficacy of sorafenib. Amentoflavone, the flavonoid compound isolated from *Selaginellat amariscina*, has been shown to enhance sorafenib-inhibited tumor growth by inducing apoptosis and reducing anti-apoptotic potential in HCC in vitro and in vivo [13,14]. PHY906 (a four-herb Chinese medicine formula) has been demonstrated to trigger anti-HCC activity of sorafenib through modulating tumor microenvironment [15]. Therefore, the combination of sorafenib and herbal medicines is a potential strategy that may be used for treatment of patients with HCC.

Magnolol, a multifunctional compound extracted from Chinese herb *Magnolia officinalis*, is able to modulate anti-inflammatory, anti-cancer, anti-oxidant, and cardio- and neuroprotective effects [15]. A previous study indicated that magnolol induced apoptosis and diminished extracellular signal-regulated kinase (ERK)-mediated metastatic potential in HCC in vitro [16]. However, whether magnolol may act as a sorafenib sensitizer that promotes anti-HCC efficacy of sorafenib is ambiguous. The purpose of the present study was to evaluate therapeutic efficacy and mechanism of sorafenib combined with magnolol in HCC in vitro and in vivo.

## 2. Results

### 2.1. Both Magnolol and LY294002 Induced Cytotoxicity of Sorafenib on SK-Hep1 and Hep3B Cells

**Figure 1:**
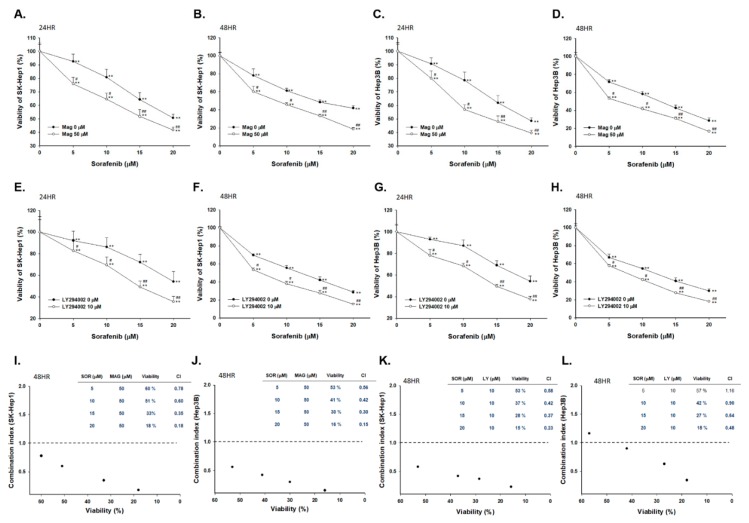
Cytotoxicity effect was enhanced in magnolol combined with sorafenib as compared to alone treatment. Viable SK-Hep1 and Hep3B cells after sorafenib (0–20 µM) combined with or without magnolol (50 µM) or LY294002 (10 µM). Cells were assayed by MTT methods and quantified by excel software. The combination results were all normalized with alone treatment of magnolol (50 µM) or LY294002 (10 µM). Cytotoxicity results of magnolol combined sorafenib for (**A**) 24 h and (**B**) 48 h on SK-Hep1 cells; or on Hep3B cells for (**C**) 24 h and (**D**) 48 h. The quantification result of sorafenib combined with or without LY294002 (10 µM) for (**E**) 24 h and (**F**) 48 h on SK-Hep1 cells; or on Hep3B cells for (**G**) 24 h and (**H**) 48 h. Combination index of sorafenib combined with (**I**,**J**) magnolol or (**K**,**L**) LY294002 for 48 h on SK-Hep1 or Hep3B cells. (** *p* < 0.01 was compared with 0 µM sorafenib; ^#^ *p* < 0.05 and ^##^ *p* < 0.01 were both compared with alone treatment).

Magnolol (50 µM) alone treatment slightly induced cytotoxicity of HCC cells, which is represented in Supplementary Figure S1. We then evaluated whether sorafenib may markedly reduce cell viability via combination with magnolol as compared with mono-treatment. As shown in Figure 1A,B, viable SK-Hep1 cells were decreased by combination treatment in a dose- and time-dependent manner. In addition, another HCC cell line, Hep3B showed similar toxicity results of magnolol combined sorafenib for 24 and 48 h (Figure 1C,D). In both HCC cells, the combination group showed superior cytotoxicity effect as compared to mono-treatment. Here, LY294002 (AKT inhibitor) was used to validate signaling transduction regulation of magnolol combined with sorafenib. The toxicity of sorafenib on SK-Hep1 and Hep3B cells was enhanced by LY294002 as increased by dose and time (Figure 1E–H). In order to select combination dosage with synergistic effect, we performed combination index analysis. Combination index below 1 was found in 10 µM sorafenib combined with 50 µM magnolol at 48 h treatment in two cell lines (Figure 1I,J). The synergism effect was also found in 10 µM LY294002 (AKT inhibitor) combined with 10 µM sorafenib treatment on SK-Hep1 and Hep3B cells (Figure 1K,L).

### 2.2. Magnolol Triggered the Dephosphorylation of AKT/mTOR/PRAS40 in Combined with Sorafenib

**Figure 2:**
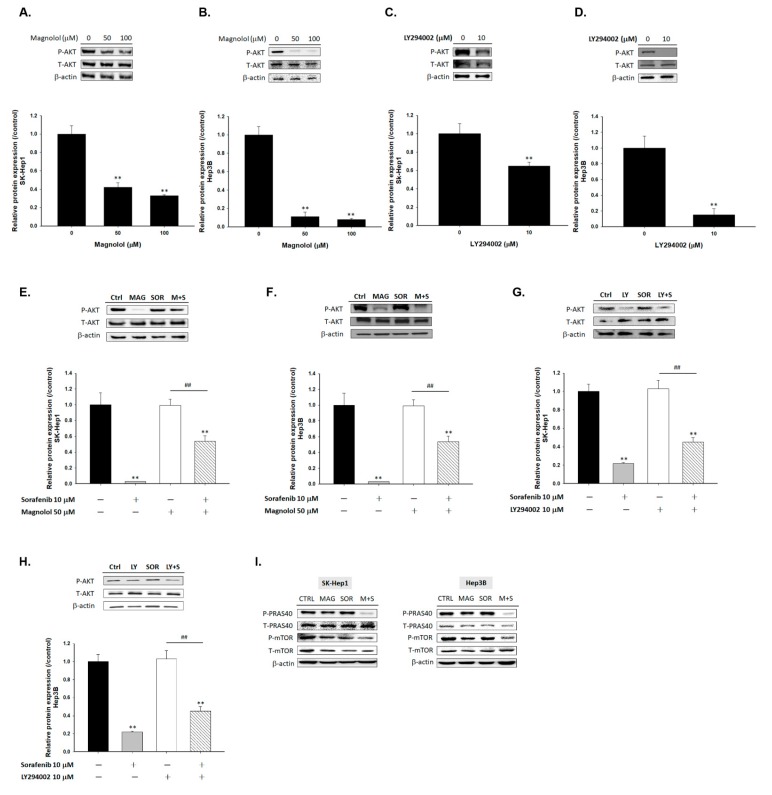
The inactivation of protein kinase B (AKT)/mTOR/PRAS40 was found in magnolol alone treatment and combination treatment group. (**A**) SK-Hep1 cells and (**B**) Hep3B cells were treated with 0, 50, 100 µM magnolol for 48 h and evaluated by Western blot. (**C**) SK-Hep1 cells and (**D**) Hep3B cells were treated with 0 or 10 µM LY294002 for 48 h and evaluated by Western blot. (**E**,**G**) SK-Hep1 cells and (**F**,**H**) Hep3B cells were treated with 10 µM sorafenib combined with 50 µM magnolol or 10 µM LY294002 for 48 h, respectively. (**I**) SK-Hep1 cells and Hep3B cells were treated with 10 µM sorafenib combined with 50 µM magnolol for 48 h (** *p* < 0.01 was compared with 0 µM sorafenib; ^##^ *p* < 0.01 were both compared with 10 µM sorafenib).

To further investigate the mechanism of magnolol induced toxicity of sorafenib on HCC cells, we performed Western blot assay. As showed in Figure 2A,B, the expression of phosphorylation AKT was significantly decreased by magnolol in SK-Hep1 or Hep3B cells. LY294002 was used as a positive control with ability to suppress the phosphorylation form of AKT. SK-Hep1 and Hep3B cells treated with AKT inhibitor (LY294002) also showed the inactivation effect on AKT (Figure 2C,D). Although AKT expression level was not affected by sorafenib alone treatment, effectively AKT inhibition was found in magnolol combined with sorafenib (Figure 2E,F). The combination of LY294002 and sorafenib showed similar AKT inhibition ability on SK-Hep1 and Hep3B cells (Figure 2G,H). Furthermore, we also validated whether magnolol combined sorafenib may affect AKT downstream proteins expression, including mTOR (mammalian target of rapamycin) and PRAS40 (proline-rich AKT substrate of 40 kDa). In Figure 2I, phosphorylation of mTOR (Ser2448) and PRAS40 (Thr246) were all decreased in magnolol alone and combination with sorafenib groups. In sum, we suggested that the enhancing toxicity of magnolol on sorafenib was mediated by AKT/mTOR/PRAS40 signaling pathway.

### 2.3. Both Magnolol and LY294002 Enhanced Sorafenib-Induced Apoptotic Cell Death and Reduced Anti-Apoptosis Proteins Expression of HCC Cells

**Figure 3:**
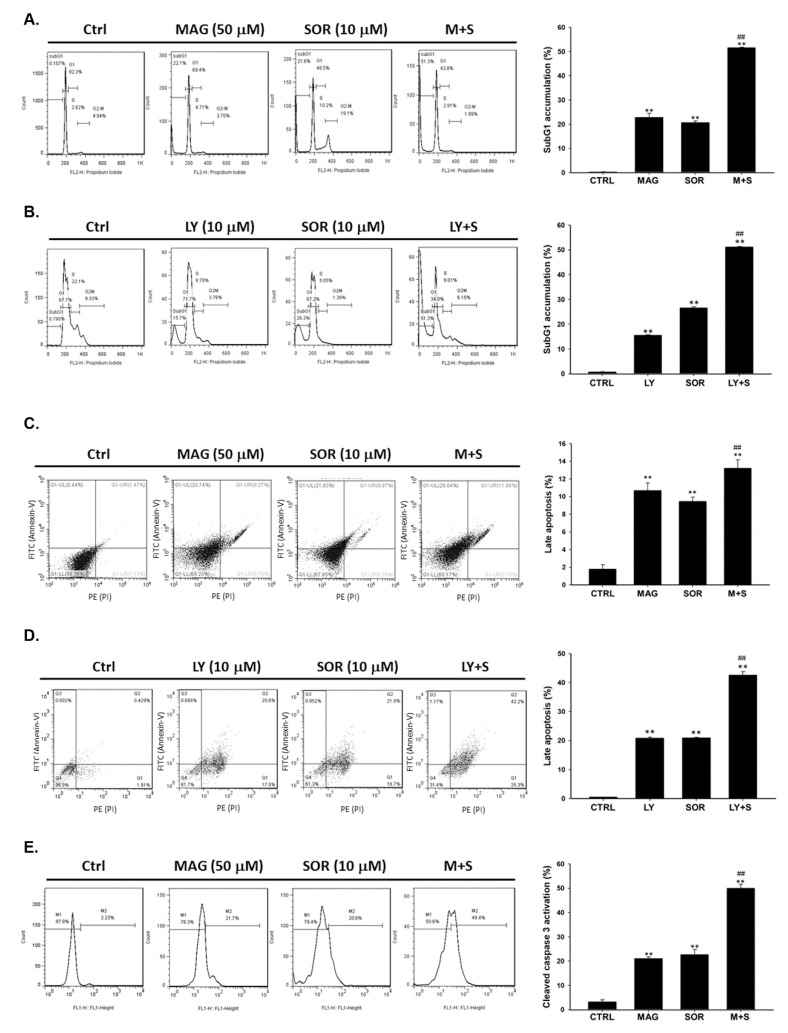

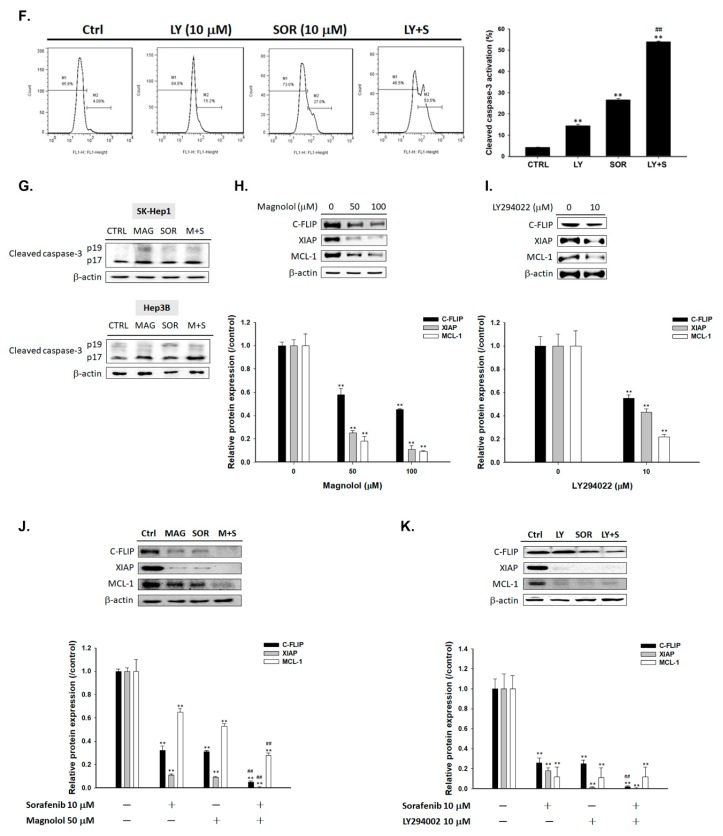
Markedly apoptotic cell death was found in magnolol co-treated sorafenib groups as compared to alone treatment. For apoptosis effect evaluation, magnolol (50 µM) or LY294002 (10 µM) combined with sorafenib (10 µM) were assayed by (**A**,**B**) cell cycle analysis, (**C**,**D**) Annexin/PI double staining, and (**E**–**G**) cleaved caspase-3 activities with flow cytometry and Western blotting, respectively. Cleaved caspase-3 expression pattern was validated on SK-Hep1 and Hep3B cells. Anti-apoptosis expressions of C-FLIP, XIAP, and MCL-1 after (**H**) magnolol alone, (**I**) LY294002 alone or combined with sorafenib and (**J**,**K**) LY294002 alone or combined with sorafenib treatment were assayed by Western blot. (** *p* < 0.01 was compared with 0 µM, ^##^ *p* < 0.01 were both compared with alone treatment).

In cell cycle analysis, subG1 phase was recognized as apoptotic cell population. We found that magnolol may increase the accumulation of subG1 population while combined with sorafenib on SK-Hep1 cells (Figure 3A). The maximal apoptotic cells number also found on LY294002 combined with sorafenib group on SK-Hep1 cells (Figure 3B). In annexin V/PI double stain experiment, a method of apoptotic cell death measurement, the increase percentage of late apoptotic cells was also observed after combination of magnolol or LY294002 with sorafenib on SK-Hep1 cells (Figure 3C,D). Furthermore, the activity of cleaved caspase-3 was also found in two type of co-treatment, including magnolol or LY294002 combined with sorafenib (Figure 3E,F). Combination of Magnolol and sorafenib also induced cleaved caspase-3 protein expression on SK-Hep1 and Hep3B cells (Figure 3G). In addition, incubation of Magnolol (Figure 3H), LY294002 (Figure 3I) alone or in combination with sorafenib abrogated the expression of the anti-apoptotic proteins C-FLIP (Cellular FLICE (FADD-like IL-1β-converting enzyme)-inhibitory protein), XIAP (X-linked inhibitor of apoptosis protein), and MCL-1 (myeloid cell leukemia 1) (Figure 3J,K). Most importantly, the greatest anti-apoptosis associated proteins inhibition was found in combination of magnolol and sorafenib. In conclude, the apoptosis cell death which induced by magnolol combined sorafenib was associated with the inhibition of AKT signaling transduction.

### 2.4. Both Magnolol and LY294002 Promoted Sorafenib-Induced Activation of Death Receptor Dependent Extrinsic Apoptosis in SK-Hep1 Cells

**Figure 4:**
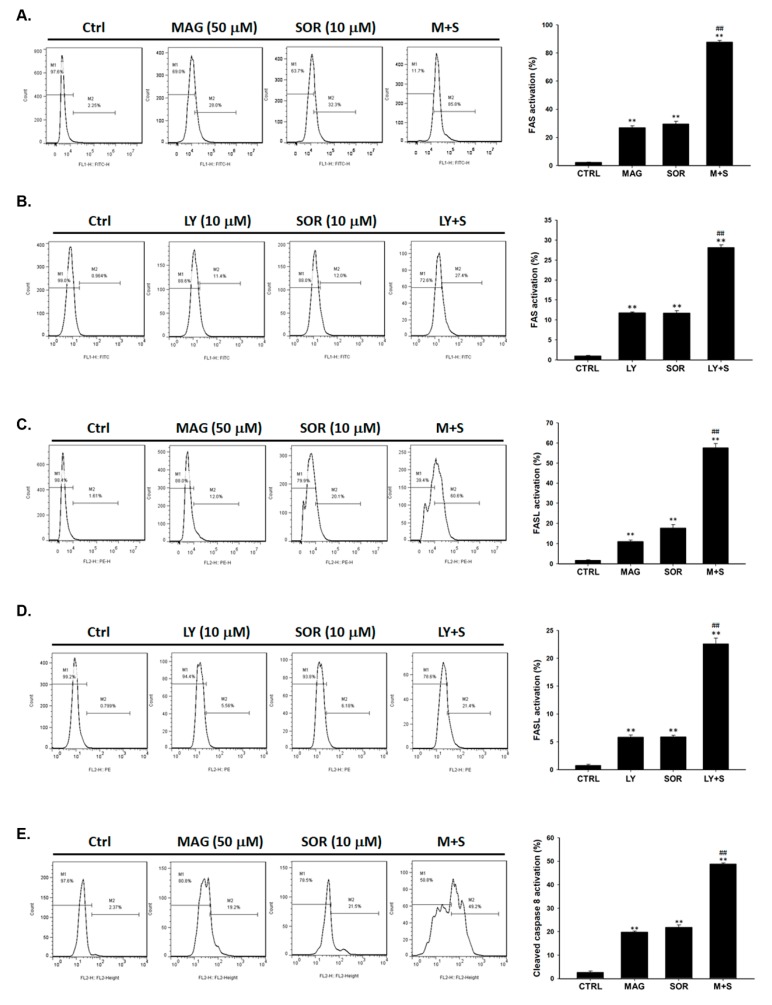

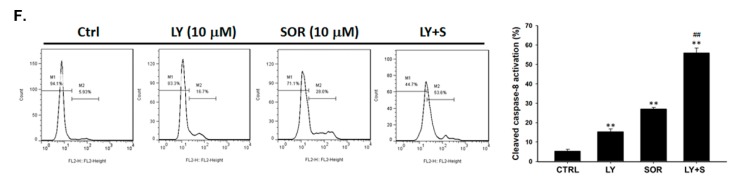
Death receptor dependent markers were markedly increased by magnolol combined with sorafenib as compared to alone treatment. SK-Hep1 cells treated with magnolol (50 µM) or LY294002 (10 µM) combined with sorafenib (10 µM) were stained for (**A**,**B**) FAS (**C**,**D**) FASL and evaluate the activation with flow cytometry. (**E**,**F**) Cleaved caspase-8 activation was evaluated by flow cytometry. (** *p* < 0.01 was compared with 0 µM, ^##^ *p* < 0.01 were both compared with alone treatment).

To validate the main controller of extrinsic apoptosis signal on the upstream, we further investigated the activation of death receptor FAS (CD95) and their related ligand FASL (CD95L). As shown in Figure 4A,B, both magnolol and LY294002 co-treated with sorafenib were markedly increased the activation of FAS as compared to alone treatment. FASL expression in these two combination groups also showed maximal increasing activity (Figure 4C,D). Furthermore, magnolol combined sorafenib enhanced the activation of cleaved caspase-8 to around 50%, which increased 30% more as compared with both type of single treatment (Figure 4E). The similar activation result of caspase-8 was found in LY294002 combined with sorafenib (Figure 4F).

### 2.5. Both Magnolol and LY294002 Triggered Sorafenib-Initiated Intrinsic Apoptosis and Cleavage of PARP-1 in HCC Cells

**Figure 5:**
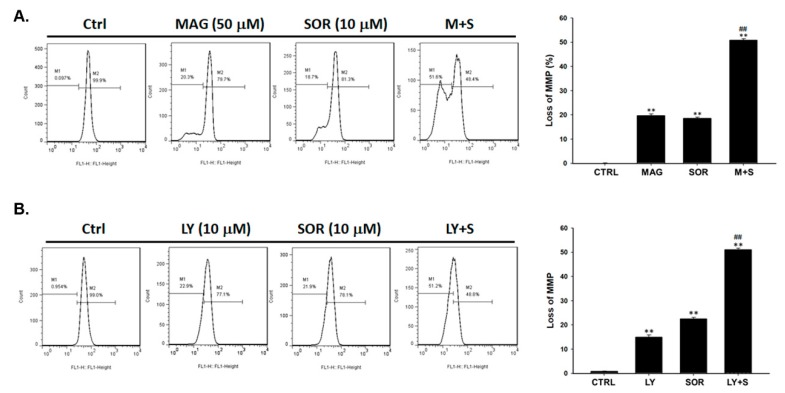

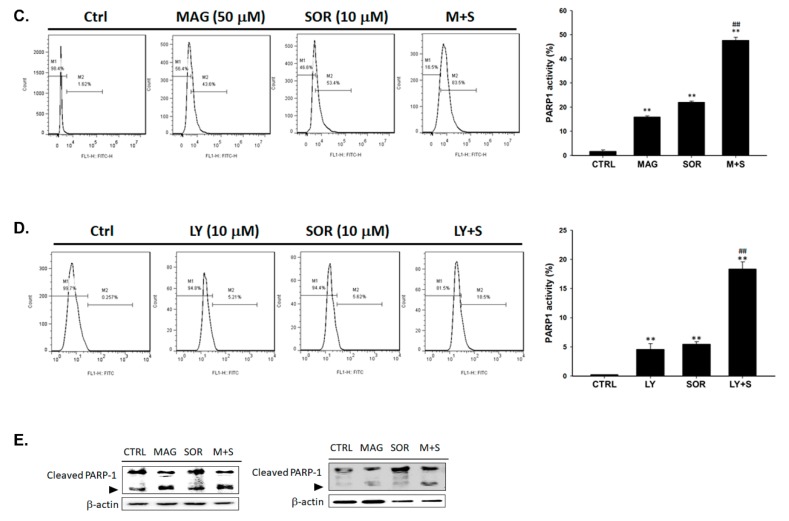
Intrinsic apoptosis and DNA damage were both obviously induced by magnolol combined with sorafenib as compared to single therapy. The loss of mitochondria potential after (**A**) magnolol (50 µM) or (**B**) LY294002 (10 µM) combined with sorafenib (10 µM) were validated by flow cytometry. The activation of cleavage PARP-1 after (**C**) magnolol (50 µM) or (**D**) LY294002 (10 µM) combine sorafenib (10 µM) were validated by flow cytometry. (**E**) Cleaved PARP-1 was validated by Western blotting on SK-Hep1 (left panel) and Hep3B (right panel) cells. (** *p* < 0.01 was compared with 0 µM, ^##^ *p* < 0.01 were both compared with alone treatment).

To investigate the apoptosis signaling within cellular, we investigated the change of intrinsic marker mitochondria potential (MMP) after magnolol or LY294002 combined with sorafenib on SK-Hep1 cells. As shown in Figure 5A, the mitochondria dependent apoptosis was significantly activated by magnolol combined sorafenib as compare to alone therapy. Through AKT blockage and combined with sorafenib, mitochondria potential was effectively decreased as magnolol combined with sorafenib (Figure 5B). These results suggested that AKT inhibition by magnolol or AKT inhibitor may effectually promote sorafenib-mediated intrinsic apoptosis mechanism in hepatocellular carcinoma cells. After validating apoptosis mechanism of magnolol combined with sorafenib, we then investigated DNA damage alteration in combination situation. We found that the activated caspase-3 by magnolol and sorafenib co-treatment may also trigger the cleavage of PARP-1 (Poly [ADP-ribose] polymerase 1) (Figure 5C). AKT inhibitor combined with sorafenib demonstrated similar results of increasing cleavage of PARP1 as similar to magnolol combined with sorafenib (Figure 5D). In addition, cleaved PARP protein expression was also increased on combination treatment of SK-Hep1 and Hep3B cells (Figure 5E). Taken together, the combination of magnolol or LY294002 showed superior intrinsic apoptosis and caspase-3 mediated PARP-1 cleavage as compare to monotherapy.

### 2.6. Both Magnolol and LY294002 Boosted Sorafenib-Reduced Migration and Invasion Ability of SK-Hep1 Cells

**Figure 6:**
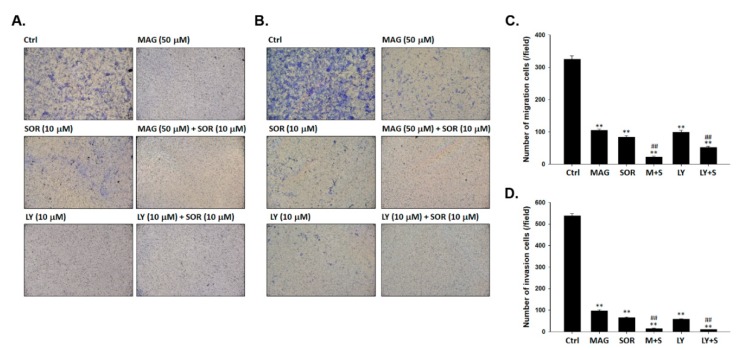
Numbers of migrated and invaded cancer cells were markedly reduced by magnolol combined with sorafenib as compared to monotherapy. The transwell assay was performed after magnolol (50 µM) or LY294002 (10 µM) combined with sorafenib (10 µM) to validate (**A**,**C**) migration and (**B**,**D**) invasion effect on SK-Hep1 cells. (** *p* < 0.01 was compared with 0 µM, ^##^ *p* < 0.01 were both compared with alone treatment).

To investigate whether magnolol may enhance the invasion and migration inhibitory effect of sorafenib in tumor cells, we performed with transwell assay. Transwells without matrigel were used as a cancer cells migration validation platform; however, coated with matrigel was used as an invasion validation platform. In Figure 6A,C, the effect of migration was suppressed by a combination of magnolol or LY294002 with sorafenib, respectively. Magnolol or LY294002 combined with sorafenib were both decreased the number of invaded cells as showed in Figure 6B,D. In conclude magnolol and sorafenib may suppress the migration and invasion of SK-Hep1 cells via inhibition of AKT signaling transduction. Markedly invasion and migration inhibition was found in combination of magnolol or LY294002 combined with sorafenib as compared to alone treatment.

### 2.7. Magnolol and Sorafenib Co-Treatment Markedly Suppressed Tumor Growth, Anti-Apoptotic Proteins Expression and Induced Apoptosis Mechanism in Sk-Hep1/luc2 and Hep3B Bearing Mice

**Figure 7:**
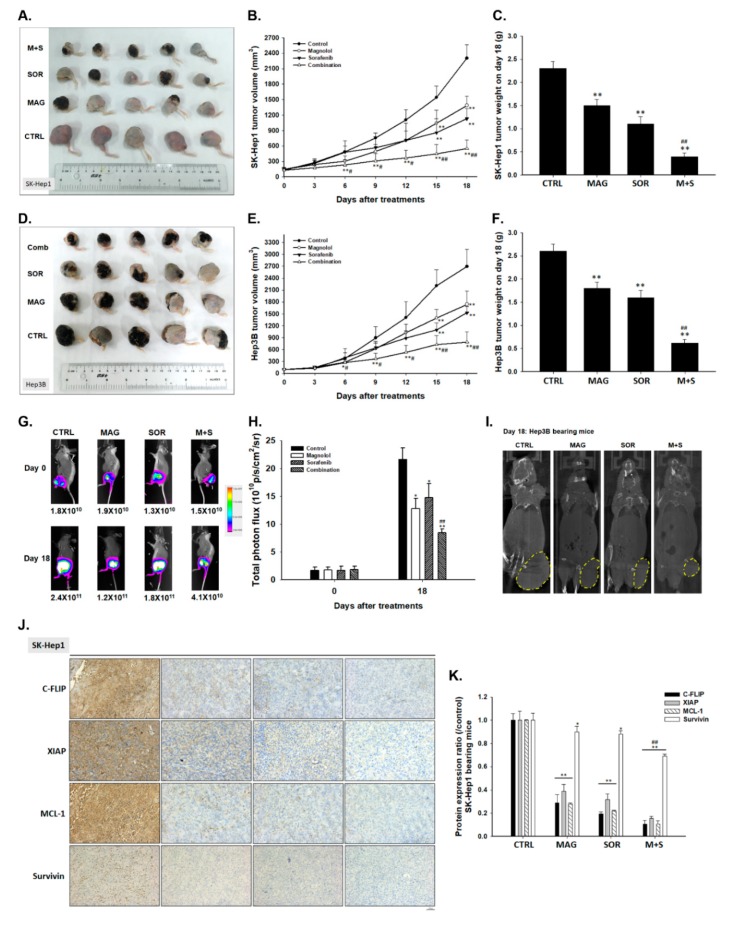

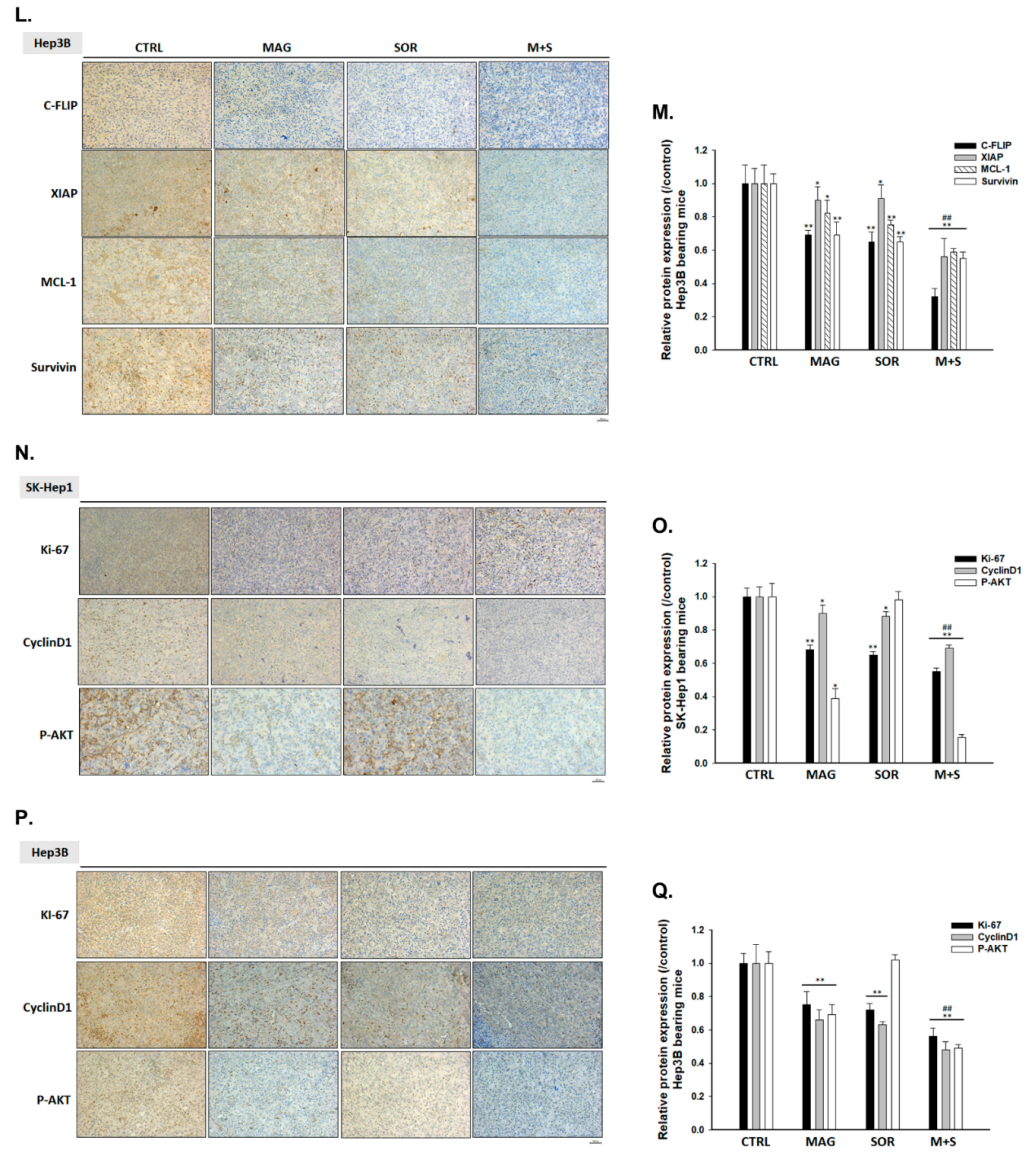

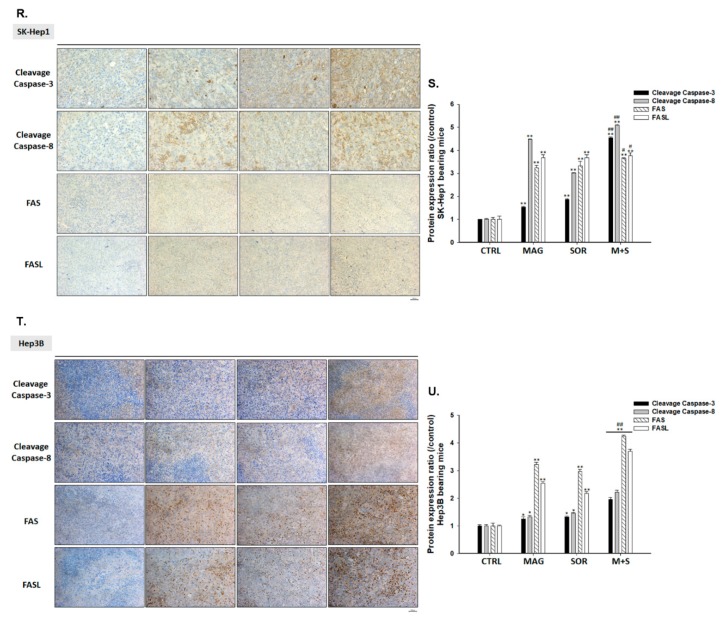
Tumor growth inhibition and apoptosis induction was found in magnolol and sorafenib co-treated mice as compare to single treatment. (**A**,**D**) Tumor tissue on left flank was displayed as a figure (n = 5). Animal experiment was repeated twice. (**B**,**E**) Tumor volume was measured every three days by caliper. (**C**,**F**) Tumor weight was validated by digital weight. (**G**) BLI on day 0 and 18 of each group on SK-Hep1*/luc2* bearing mice was displayed as a figure. (**H**) Total photon flux (p/s/cm^2^/sr) from each group was quantified. (**I**) CT images on day 18 of each group on Hep3B bearing mice was displayed as a figure. Immunohistochemically (IHC) staining of (**J**–**M**) anti-apoptosis related proteins, (**N**–**Q**) proliferation proteins, and (**R**–**U**) apoptosis related proteins on SK-Hep1*/luc2* or Hep3B bearing mice. (** *p* < 0.01 was compared with control, ^##^ *p* < 0.01 were both compared with alone treatment).

To determine the anti-tumor ability of magnolol combined with sorafenib, we evaluated tumor volume change, body weight, anti-apoptosis related, and apoptosis related proteins levels on Sk-Hep1/*luc2* and Hep3B bearing mice. As shown in Figure 7A,D, the smallest tumor size was found in combination group as compared to control and single treatment. Quantification tumor volume results in Figure 7B,E also demonstrated the greatest tumor growth inhibition in magnolol and sorafenib co-treatment group. Tumor extracted on day 18 also demonstrated the decreasing tumor weight in the combination group (Figure 7C,F). Furthermore, magnolol combined with sorafenib reduced the BLI signal intensity (Figure 7G) as showed in image and the quantification photon flux (Figure 7H) from Sk-Hep1/*luc2* tumor. Computer tomography (CT) results displayed the smallest Hep3B tumor size was found in magnolol combined with sorafenib group (Figure 7I). Additionally, anti-apoptotic related MCL-1, C-FLIP, XIAP, and Survivin were all decreased by combination treatment in SK-Hep1 and Hep3B tumors (Figure 7J–M). Moreover, proliferation related proteins such as Ki-67 and CyclinD1 were all decreased in combination group (Figure 7N–Q). Interestingly, obviously P-AKT suppression was found in combination tumor tissue but not in sorafenib alone tissue (Figure 7N–Q). On the contrary, cleaved caspase-3 and caspased-8 apoptosis protein levels were both increased by combination group (Figure 7R–U). In sum, the effective anti-tumor effect of magnolol combined with sorafenib was regulated through enhancing apoptosis mechanism and suppressing AKT mediated oncogene mechanism of HCC.

### 2.8. Magnolol and Sorafenib Co-Treatment may not Trigger Liver and General Toxicity of Sk-Hep1/luc2 and Hep3B Bearing Mice

**Figure 8:**
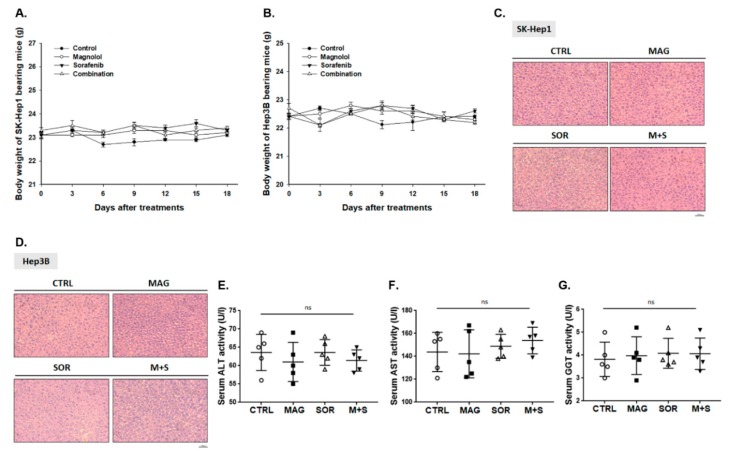
No general and liver toxicity was found in combination groups. (**A**,**B**) Body weight and (**C**,**D**) liver pathology of *Sk-Hep1/luc2* and *Hep3B* bearing mice were displayed. The value of (**E**) ALT, (**F**) AST and (**G**) γ-glutamyl transferase (GGT) from Hep3B bearing mice serum on day 18 was presented. (ns was recognized as non-significant difference).

Then, we further investigated whether combination treatment may induce general or liver toxicity effect on tumor bearing mice. Importantly, no body weight (Figure 8A,B) and liver pathology (Figure 8C,D) changes were found in all treatment groups. In addition, we also validated alanine, aspartate aminotransferase (ALT, AST), and γ-glutamyl transferase (GGT) from Hep3B bearing mice serum after sacrificed. As shown in Figure 8E–G, no significant difference was found between the control, alone treatment, and combination groups on day 18.

## 3. Discussion

Both ERK and AKT/protein kinases B (PKB) signaling were overexpressed in human HCC [17,18,19]. ERK is phosphorylated by RAF/mitogen-activated protein/ERK kinase (MEK) pathway. ERK phosphorylation promotes tumor progression through triggering activation of downstream kinases and transcription factors [20]. AKT phosphorylation is mediated with phosphatidylinositol-3-OH-kinase (PI3K) and involved in cell survival response [21]. High expression of phospho-ERK (pERK) is recognized as the predictive factors for poor survival in patients with HCC [22].

Although sorafenib inhibits tumor growth by regulating RAF/MEK/ERK pathway; PI3K/AKT signaling transduction is not affected by sorafenib treatment in HCC [23]. AKT phosphorylation mediates acquired resistance to sorafenib and inhibition of AKT phosphorylation enhances therapeutic efficacy of sorafenib in HCC [24,25]. ARQ 092, the AKT inhibitor, has been shown to promote sorafenib-inhibited tumor progression by inducing apoptosis and suppressing proliferation and angiogenesis [26]. Llerena et al. also mentioned that AKT may be a potential target for identifying sorafenib combination strategy [27]. In our results, we demonstrated that LY294002 (AKT inhibitor) and magnolol both significantly enhances sorafenib-induced cytotoxicity and apoptosis in SK-Hep1 and Hep3B cells (Figure 1). Since AKT plays a key role of sensitization tumors to sorafenib, we found that magnolol showed potential to suppress the phosphorylation of AKT in HCC (Figure 2). Furthermore, AKT activation contributes to evasion of apoptosis through inducing anti-apoptotic proteins such as C-FLIP, XIAP, and MCL-1 inhibited apoptotic signaling transduction in cancer cells [28,29,30,31]. High protein levels of C-FLIP, XIAP, and MCL-1 is involved in blockage of extrinsic/intrinsic apoptotic pathways and correlated with poor survival in patients with HCC [32]. Both suppression of C-FLIP and MCL-1 has been indicated to overcome sorafenib resistance in HCC [33,34]. Here, we indicated LY294002 or magnolol combined with sorafenib can effectively reduce anti-apoptotic protein levels of C-FLIP, XIAP, MCL-1, and Survivin (Figure 3J–M) and trigger sorafenib-induced apoptosis through extrinsic apoptosis (Figure 3 and Figure 4).

Through literature review, we found that many herbal compounds as AKT inhibitors may potentiate anti-cancer efficacy of sorafenib in HCC. Wan et al. presented tetrandrine, an alkaloid extracted from the Chinese medicinal herb, enhanced antitumor activity of sorafenib in HCC. Inhibition of AKT activation was involved in mitochondrial apoptosis induced by the combination of sorafenib with tetrandrine [35]. In our studies, we indicated that magnolol may enhance mitochondria dependent intrinsic apoptosis pathways of sorafenib (Figure 5). Bufalin, the potent antitumor compound isolated from medicine herbs, has been reported to boost anti-angiogenic effect of sorafenib via suppression of AKT/vascular endothelial growth factor (VEGF) signaling in HCC [36]. Moreover, mechanistic target of rapamycin (mTOR), serine/threonine kinase activated by PI3K/AKT pathway, regulates tumor cell growth, survival, and metabolism via initiating downstream signaling network. Activation of mTOR was overexpressed and associated with poor survival in HCC [37]. The proline-rich AKT substrate of 40 kDa (PRAS40), the substrate of AKT phosphorylated by active-AKT, promotes tumor progression through upregulating cell proliferation, anti-apoptosis, and metastasis ability [38]. The inhibitor of AKT signaling reduced activation of AKT signaling-related proteins such as mTOR, PRAS40, and ribosomal protein S6 kinase (S6K1) in HCC [39]. Li et al. demonstrated blockage of PI3K/AKT/mTOR signaling potentiated anti-HCC efficacy of sorafenib. BEZ235, inhibitor of PI3K/AKT/mTOR signaling also enhanced sorafenib-inhibited migration ability of HCC [40]. In addition to AKT dephosphorylation, Figure 2I shown magnolol also enhanced sorafenib-inhibited expression of p-mTOR and p-PRAS40 in HCC SK-Hep1 and Hep3B cells. While in Figure 6 results, we found that both magnolol and LY294002 obviously promoted sorafenib-diminished invasion and migration effect of HCC.

In this study, we found magnolol not only reduced protein levels of AKT (Ser 473) but also promoted sorafenib-inhibited tumor growth in HCC in vitro and in vivo (Figure 7). Sorafenib-induced apoptosis was facilitated by magnolol treatment through extrinsic and intrinsic apoptotic signaling transduction (Figure 4 and Figure 5). In addition, magnolol enhanced sorafenib-inhibited expression of proliferation, anti-apoptotic, and cell invasion related proteins (Figure 6).

## 4. Methods

### 4.1. Drugs and Chemical Reagents

Sorafenib was kindly provided by Bayer Corporation (Whippany, NJ, USA). Magnolol, MTT (3-(4,5-Dimethylthiazol-2-yl)-2,5-Diphenyltetrazolium Bromide), RNase, and dimethyl sulfoxide (DMSO) were purchased from Sigma Chemical Co. (St. Louis, MO, USA). LY294002, an AKT inhibitor, was purchased from Selleckchem (Houston, TX, USA).

### 4.2. Cell Culture

The SK-Hep1 and Hep3B human hepatocellular carcinoma cell line was kindly provided from prof. Jing-Gung Chung, China Medial University, Taichung, Taiwan. Cells were placed into 10 cm^2^ culture dish and grown at 37 °C under a humidified 5% CO_2_ atmosphere in Dulbecco’s Modified Eagle Medium (DMEM) medium with 2 mM L-glutamine, 10% fetal bovine serum, 100 Units/mL penicillin, and 100 mg/mL streptomycin. All cell culture related products were purchased from GIBCO^®^/Invitrogen Life Technologies (Carlsbad, CA, USA) [41].

### 4.3. Transfection and Stable Clone Selection

Transfection procedure was followed by our previous studies [42]. In brief, SK-Hep1 cells were transfected with pGL4.50 luciferase reporter (pGL4.50 [luc2/CMV]) (Promega. Madison, WI, USA). Stable clone with luc2 reporter gene expression was selected by 200 μg/mL hygromycin for 2 weeks and monitored by IVIS100 Imaging System (Xenogen, Alameda, CA, USA).

### 4.4. Determinations for Viable Cells

MTT (3-(4,5-dimethylthiazol-2-yl)-2,5-diphenyltetrazolium bromide) assay was used as a viable cell validation method. SK-Hep1 cells at a density of 3 × 10^4^ cells/well were placed in 96-well plates for 24 h and were treated with 0–20 µM of sorafenib, 0–200 µM magnolol and combine with or without 50 µM magnolol or 10 µM LY294002 for 24 h and 48 h before cells stained by MTT as described previously [43]. To select appropriate synergistic combination dosage of two drugs, CompuSyn software (ComboSyn, Paramus, NJ, USA) was used to calculate the CI-isobologram, which was developed by Chou and Talalay [44,45]. CI = 1, additive effect; CI < 1, synergistic effect; CI > 1, antagonistic effect.

### 4.5. Measurements of Apoptotic Cell Death

SK-Hep1 (5 × 10^5^ per well) cells were maintained in 6-well plates and were incubated with 10 µM sorafenib, 10 µM LY294002, 50 µM magnolol, sorafenib plus magnolol, sorafenib plus LY294002, respectively, for 48 h. At the end of incubation, cells were isolated, fixed with 70% EtOH, stained with Propidium Iodide (PI, Sigma Chemical Co.) and then were analyzed by flow cytometry as described previously, while the distribution of cell cycle sub-G1 phase represented as apoptosis was determined [13]. In addition, Annexin V-FITC apoptosis detection kit (Vazyme Biotech Co., Ltd., Nanjing, China) was used for measuring the apoptotic cell death that are stained with Annexin-V (FITC), the necrotic cells are stained with PI (Propidium iodide) (PE) and live cells were not stained by both dyes as described previously. Cells from each treatment were isolated and were resuspended in Annexin V binding buffer, followed by incubation with Annexin V-FITC/PI in the dark for 15 min as per the manufacturer’s instructions for labeling of apoptotic cells. About 10,000 cells from each treatment were analyzed using BD FACS Calibur flow cytometry (BD Biosciences, FACS Calibur, San Jose, CA, USA). Experiments were performed in triplicate. All staining was analyzed at the BD FACS Calibur flow cytometry with FlowJo software (version 7.6.1; FlowJo LLC, Ashland, OR, USA) [46,47].

### 4.6. Measurements of Mitochondrial Membrane Potential (MMP, ΔΨm) in SK-Hep1 Cells

Flow cytometric assay was used for measuring the production levels of ΔΨm. DiOC_6_ was bought from Enzo Life Sciences (Farmingdale, NY, USA). SK-Hep1 (5 × 10^5^ per well) in 6-well-plate were treated with 10 µM sorafenib, 10 µM LY294002, 50 µM magnolol, sorafenib plus magnolol or sorafenib plus LY294002, respectively, for 48 h. After incubation, cells were harvested and re-suspended in 500 µL of DiOC_6_ (4 µM) for 30 min to measure the changes ΔΨm levels. All samples were analyzed by flow cytometry as described previously [48,49].

### 4.7. Measurements of Caspase-3 and -8 Activities

SK-Hep1 (5 × 10^5^ per well) cells were maintained in 6-well plates for one day and treated with 10 µM sorafenib, 10 µM LY294002, 50 µM magnolol, sorafenib plus magnolol, sorafenib plus LY294002, respectively, for 48 h. Cells were then collected and re-suspended in 300 µL of 1 µL substrate solutions (fluorescein isothiocyanate-Asp(OCH3)-Glu(OCH3)-Val-Asp(OCH3)-fluoromethyl ketone (FITC-DEVD-FMK) and sulforhodamine-Ile-Glu-Thr-Asp-fluoromethyl ketone (Red-IETDFMK), respectively of caspase-3 and -8 for measuring the activity of the individual caspase using flow cytometry as previously described [48]. CaspGlow fluorescein active Caspase-3 and -8 staining kit was acquired from BioVision (Milpitas, CA, USA). Quantification was measured by FlowJo software (version 7.6.1).

### 4.8. Measurements of FAS and FAS-L Activities

SK-Hep1 (5 × 10^5^ per well) cells were seeded in 6-well plates. After treated with 10 µM sorafenib, 10 µM LY294002, 50 µM magnolol, sorafenib plus magnolol or sorafenib plus LY294002 for 48 h, cells were harvested for detection of FAS and FASL activities. Cells were washed in 1% FBS/PBS and then FITC-conjugated hamster anti-mouse Fas antibody and PE-conjugated hamster anti-mouse FasL antibody were added (Thermo Fisher Scientific). All samples were analyzed by flow cytometry and FlowJo software 7.6.1 as described previously [48].

### 4.9. Measurements of Cleaved PARP-1 Activities

PARP-1 FITC apoptosis kit was purchased from Thermo Fisher Scientific, which contain with PARP-1 cleavage antibody-FITC conjugated. Flow cytometry detection of c-PARP was carried out following the kit instructions. SK-Hep1 (5 × 10^5^ per well) cells were maintained in 6-well plates then incubated with 10 µM sorafenib, 10 µM LY294002, 50 µM magnolol, sorafenib plus magnolol, sorafenib plus LY294002, respectively, for 48 h. Cells were then harvested, washed, fixed with 4% formaldehyde in ice for 15 min, double-washed with PBS and permeabilized in 90% ice methanol overnight. Next day, samples were washed and re-suspended in PBS with 1 µL of PARP-1-FITC antibody for 1 h. Flow cytometry analyses were carried out with a BD FACS Calibur flow cytometry with semiconductor lasers emitting at 405 and 488 nm. The analysis of the flow cytometry data was carried out using FlowJo software 7.6.1 [50].

### 4.10. Measurements of Protein Expression by Western Blotting Analysis

For the detection of anti-apoptosis-inducing protein, SK-Hep1 or Hep3B cells (3 × 10^6^) were placed in 10 cm dish and then were incubated with 10 µM sorafenib, 10 µM LY294002, 50 µM magnolol, sorafenib plus magnolol, sorafenib plus LY294002, respectively, for 48 h. At the end of incubation, cells were harvested, washed twice with PBS and then lysed in a buffer containing 50mM Tris-HCl (pH 8.0), 120 mM NaCl, 0.5% Nonide P-40 and 1% phosphatase inhibitor mixture II (Sigma-Aldrich Corp.) The lysated were kept on ice for 2 h and centrifugated 13,000 rpm for 15 min at 4 °C. The supernatant was quantitated the total protein by using Pierce BCA protein assay kit (Thermo Fisher Scientific). The cell lysates (40–60 µg of each) were separated by SDS-PAGE on a polyacrylamide gel followed by electrotransferred onto a PVDF membrane (EMD Millipore, Burlington, MA, USA). The membranes were incubated with blocking buffer of 5% FBS in tris-buffered saline containing 0.2% Tween 20 for 1 h at 25 °C. The blots were then gently shake with primary antibodies (1:1000 dilutions in blocking buffer) overnight at 4 °C. After being washed, secondary antibodies were applied (dilution of 1:10,000) in blocking buffer for 1 h at room temperature. Horseradish peroxidase (HRP) conjugated goat anti-rabbit or anti-mouse IgG was used as a secondary antibody for enhanced Chemiluminescent HRP Substrate (Pierce, Rockford, IL, USA) [32,49]. The primary antibodies and secondary antibodies were purchased from different companies as described as follows: C-FLIP (Cell signaling Technology, Danvers, MA, USA), MCL-1 (BioVision, Milpitas, CA, USA), XIAP, Ki-67, CyclinD1 (Thermo Fisher Scientific, Fremont, CA, USA), Survivin, P-MTOR(Ser2448), MTOR (Elabscience Biotechnology Inc., Houston, TX, USA), Phospho-Akt(Ser473), P-PRAS40(Thr246), PRAS40, PARP (Cell signaling), T-AKT (Santa Cruz Biotechnology, Inc., Dallas, TX, USA) and β-actin (Santa Cruz). Peroxidase AffiniPure Goat Anti-Mouse IgG and Goat Anti-Rabbit IgG were purchased from Jackson ImmunoResearch (West Grove, PA, USA).

### 4.11. Measurements of Migration Ability by Transwell Assay

Cell migration assay was used for evaluation of cell migration. SK-Hep1 cells (3 × 10^6^) were placed in 10 cm dish and then were incubated with 10 µM sorafenib, 10 µM LY294002, 50 µM magnolol, sorafenib plus magnolol, or sorafenib plus LY294002, respectively, for 48 h. After treatments, cells were harvested by centrifugation and 1 × 10^6^ cells were resuspended in l mL serum free medium. Two hundred µL cell suspension was added into upper chamber of transwell insert with 8 µm pore size and 500 µL DMEM with 10% FBS was added into lower chamber of transwell insert. Transwell inserts were incubated in 37 °C for 24 h. The migrating cells were fixed with 4% formaldehyde in PBS for 15 min and then stained with 0.5% crystal violet for 15 min, counted and photographed under a light microscope at ×100 [49].

### 4.12. Measurements of Invasion Ability by Transwell Assay

Cell invasion assay was used for evaluation of cell invasion. SK-Hep1 cells (3 × 10^6^) were placed in 10 cm dish and then were incubated with 10 µM sorafenib, 10 µM LY294002, 50 µM magnolol, sorafenib plus magnolol, or sorafenib plus LY294002, respectively, for 48 h. Transwell insert with 8 µm pore size was coated with 50 µL matrigel and incubated for 24 h. After treatments, cells were harvested by centrifugation and 1 × 10^6^ cells were resuspended in l mL serum free medium. Two hundred µL cell suspension was added into upper chamber of transwell insert with 8 µm pore size and 500 µL DMEM with 10% FBS was added into lower chamber of transwell insert. Transwell inserts were incubated in 37 °C for 24 h. The invasive cells were fixed with 4% formaldehyde in PBS for 15 min and then stained with 0.5% crystal violet for 15 min, counted and photographed under a light microscope at 100× [49].

### 4.13. Measurements of Tumor Growth In Vivo by Caliper, Computer Tomography and Bioluminescent Imaging (BLI)

All animal experiments were approval by Institutional Animal Care and Use Committee in Taipei Medical University (certification number: LAC-2018-0026). Eight-week-old CAnN.Cg-Foxn1nu/CrlBltw nude mice were purchased from Taiwan National Laboratory Animal Center. 1 × 10^7^ SK-Hep1/*luc2* and Hep3B cells were subcutaneous inoculated into mice left flank and allowed tumor growth for two weeks. After tumor size reached 100 mm^3^, mice were randomly separated into four different groups as follow: control (Vehicle treated with 0.1% DMSO in 100 µL PBS per day by gavage), magnolol (100 mg/kg in 100 µL PBS per day by gavage), sorafenib (10 mg/kg in 100 µL PBS per day by gavage), and combination treatment. For bioluminescent imaging, SK-Hep1/*luc2* bearing mice were anesthetized with 1–3% isoflurane and i.p. injected with 150 mg/kg D-luciferin 10 min before image acquisition. The emitted photon was acquired by Xtreme (Bruker, Billerica, MA, USA) on day 0 and 15, then quantified by Molecular Imaging Software (MI) version 7.2 (Bruker). After anesthetized, Hep3B bearing mice were performed computer tomography (Mediso Ltd., Budapest, Hungary) scanning on day 18 after treatment [51]. Mice were finally sacrificed on day 18 and prepared for further experiment.

### 4.14. Measurements of Tumor Anti-Apoptosis and Apoptosis Protein Expression

Protein levels in tumor tissue were assayed by immunohistochemically (IHC) staining. The procedure was followed by the instructions which provided from commercial kit (EMD Millipore). Sliced were stained by various primary antibodies at the indicated dilutions: MCL-1 (1:200), C-FLIP (1:200), XIAP (1:200), Survivin (1:200), Ki-67 (1:400), CyclinD1 (1:400), P-AKT (1:400), caspase-3 (1:300), and caspase-8 (1:300) 4 °C overnight and followed with secondary antibody for 30 min. Finally, the stained slides were scanned using the microscopy-based TissueFAXSp latform (TissueGnostics, Vienna, Austria) and images were captured at 100× magnification [52]. The positive protein expression levels from 5 different fields of each group were quantified by ImageJ software version 1.50 (National Institutes of Health, Bethesda, MD, USA).

### 4.15. Measurements of General Toxicity

General toxicity of treatment was determined by body weight and H&E liver stain. Mice body weight was acquired by digital weight on day 0, 3, 6, 9, 12, 15, and 18. Mice liver were extracted on day 18 and prepared for H&E stain. H&E stained methods was described in detail in our previous studies [42]. Mice serum was collected on day 18 for liver enzymes γ-glutamyl transferase (GGT), alanine and aspartate aminotransferase (ALT, AST) analysis.

### 4.16. Statistical Analysis

The experimental data were expressed as mean standard deviation (mean  ±  SD), and analyzed by excel 2017 version with Student’s *t*-test method. The experiment was repeated at three times. *p*  < 0.05 and *p*  <  0.01 were notably significant difference and datum was considered statistically significant.

## 5. Conclusions

In conclusion, magnolol enhances anti-HCC efficacy of sorafenib through inactivation of AKT activation. We suggested the combination of sorafenib with magnolol as the potential strategy may render therapeutic benefits for patients with HCC.

## Supplementary Materials

The following are available online at https://www.mdpi.com/2072-6694/12/1/87/s1, Figure S1: Cytotoxicity of SK-Hep1 and Hep3B were increased by 48 h magnolol treatment. Figure S2: Full blot images of Western blotting from Figure 2, Figure 3 and Figure 5 in main text.
