# Supplementary material for: Protein Kinase B Inactivation Is Associated with Magnolol-Enhanced Therapeutic Efficacy of Sorafenib in Hepatocellular Carcinoma In Vitro and In Vivo
Source: Cancers (Basel). 2019 Dec 30;12(1):87. doi: 10.3390/cancers12010087 (PMC7017147; doi:10.3390/cancers12010087)
Supplement: Supplementary file 1 [file cancers-12-00087-s001.pdf]

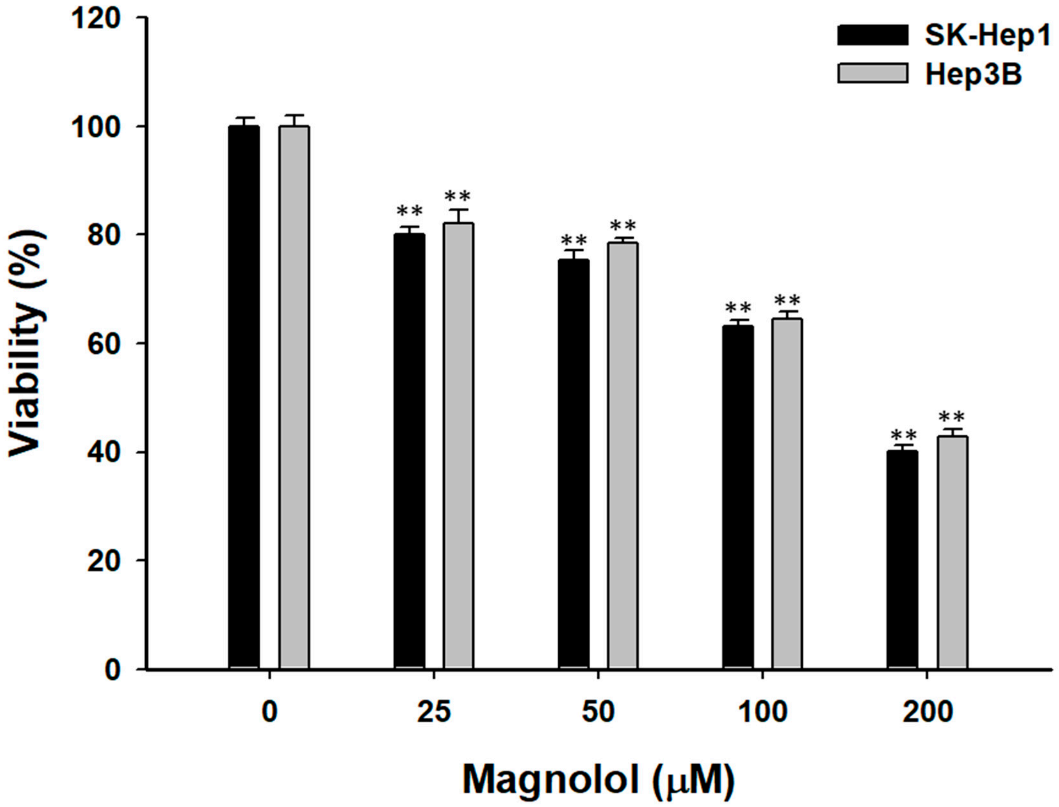

Figure 1. Cytotoxicity of SK-Hep1 and Hep3B were increased by 48 h magnolol treatment.

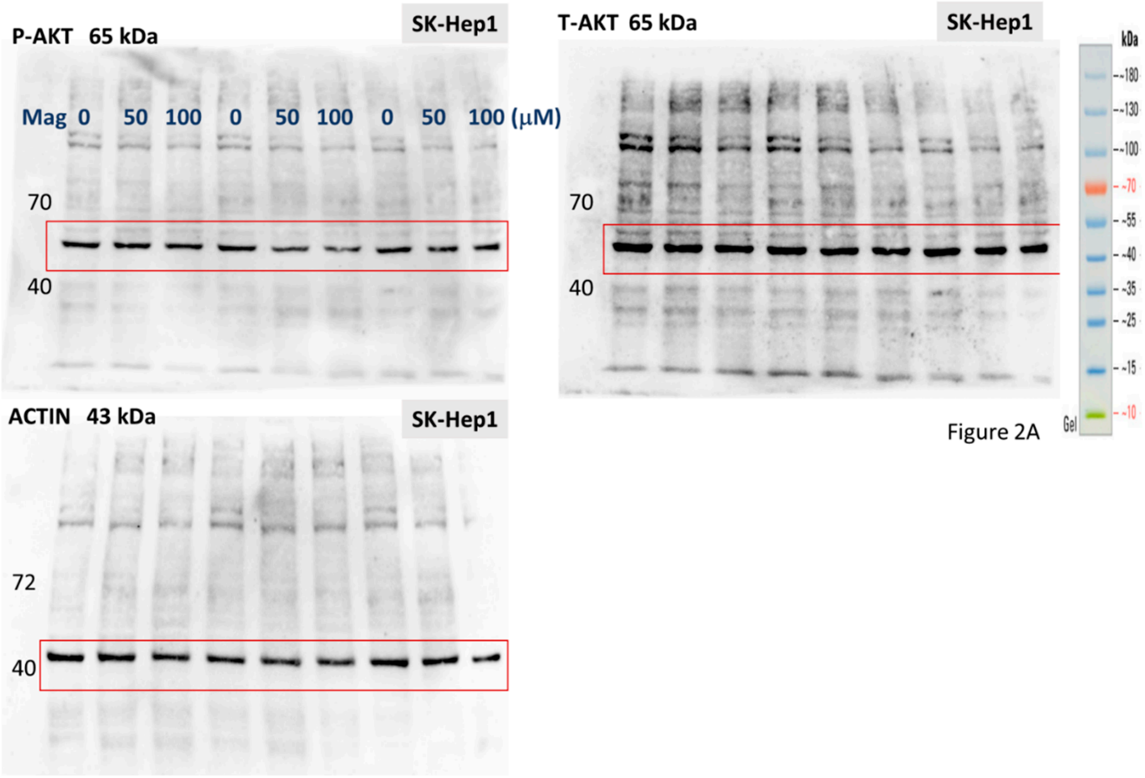

Figure 2A

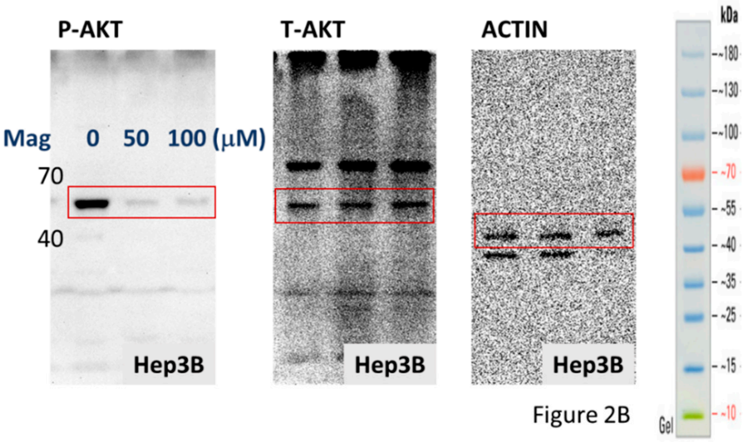

Figure 2B

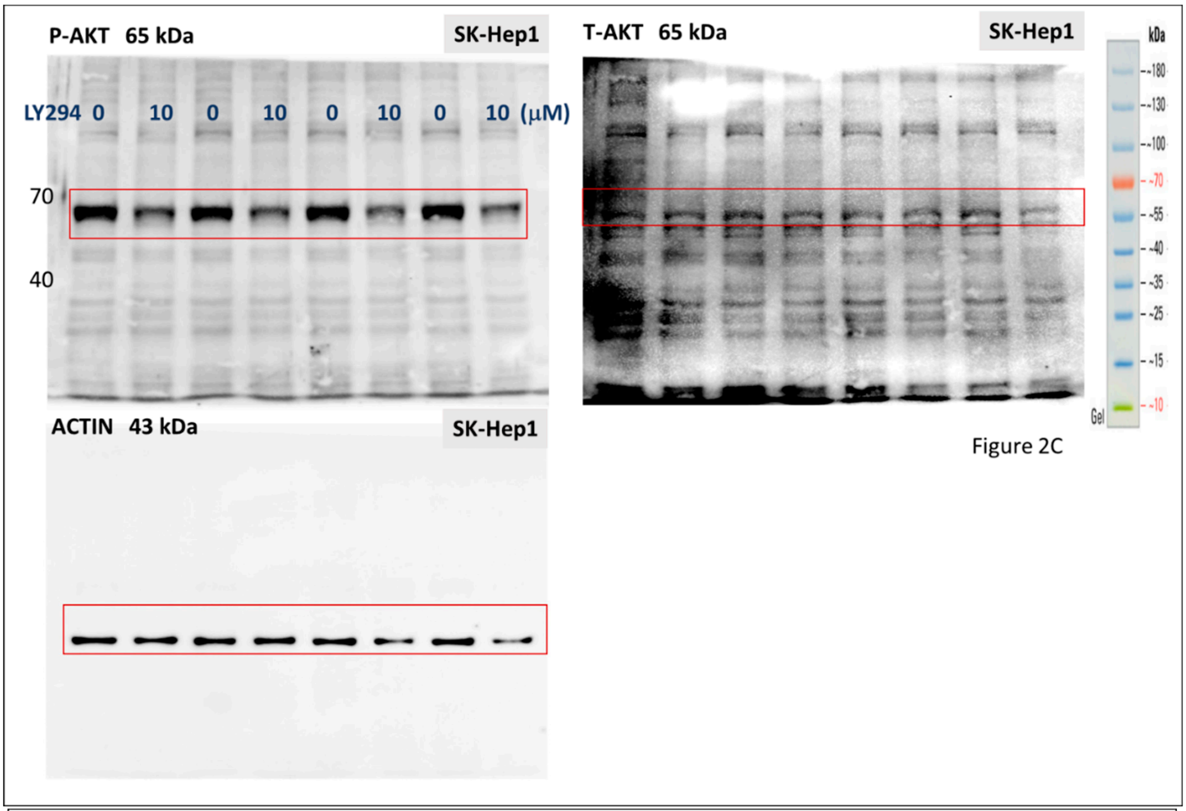

Figure 2C

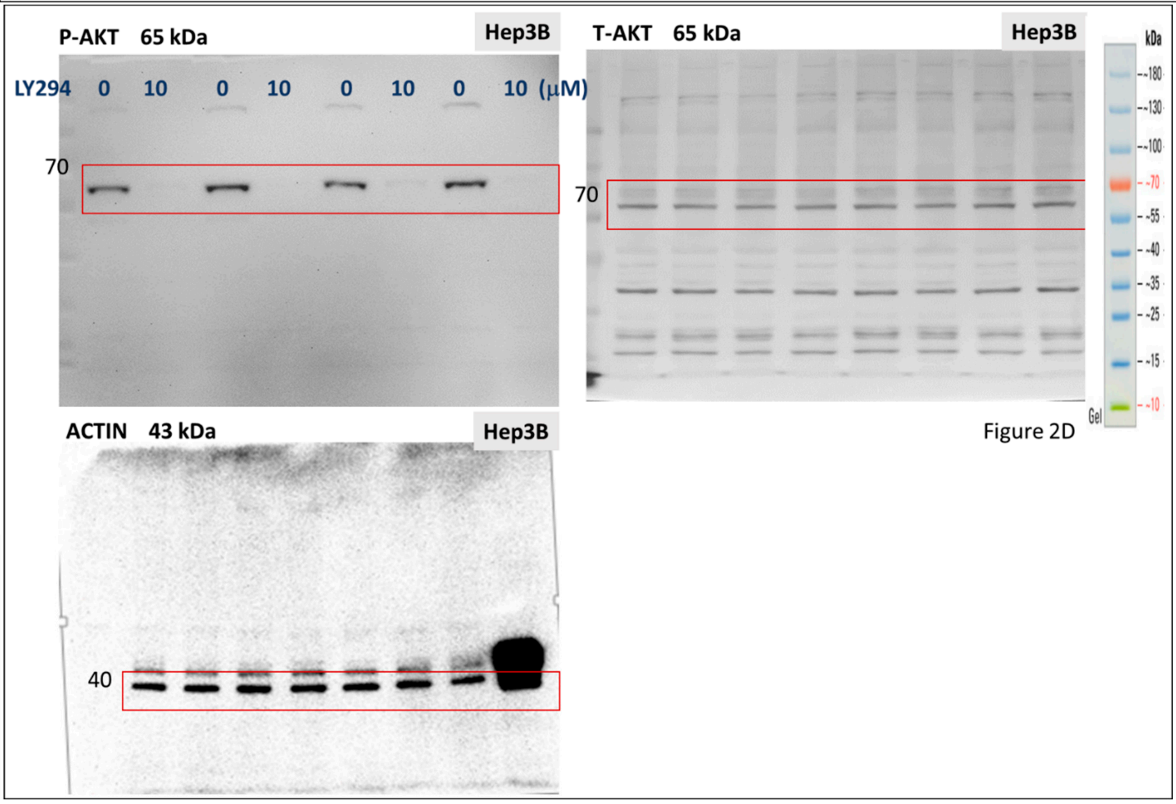

Figure 2D

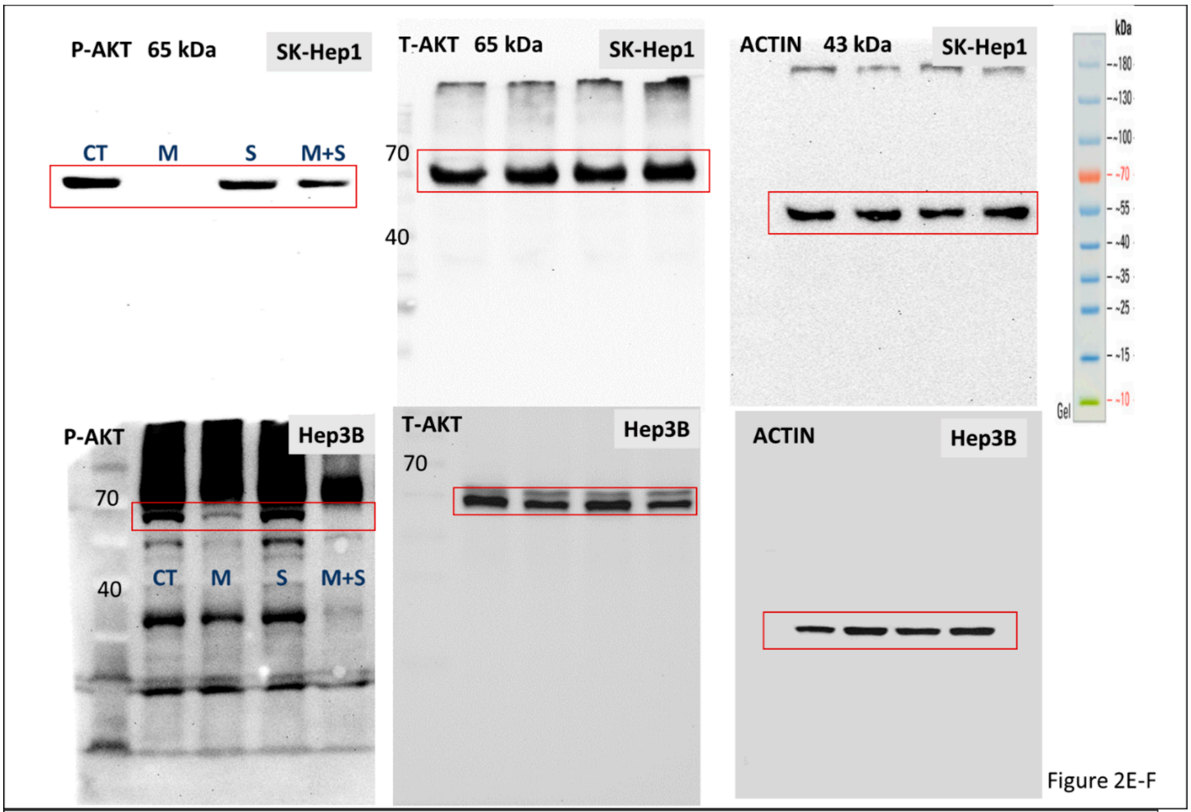

Figure 2E-F

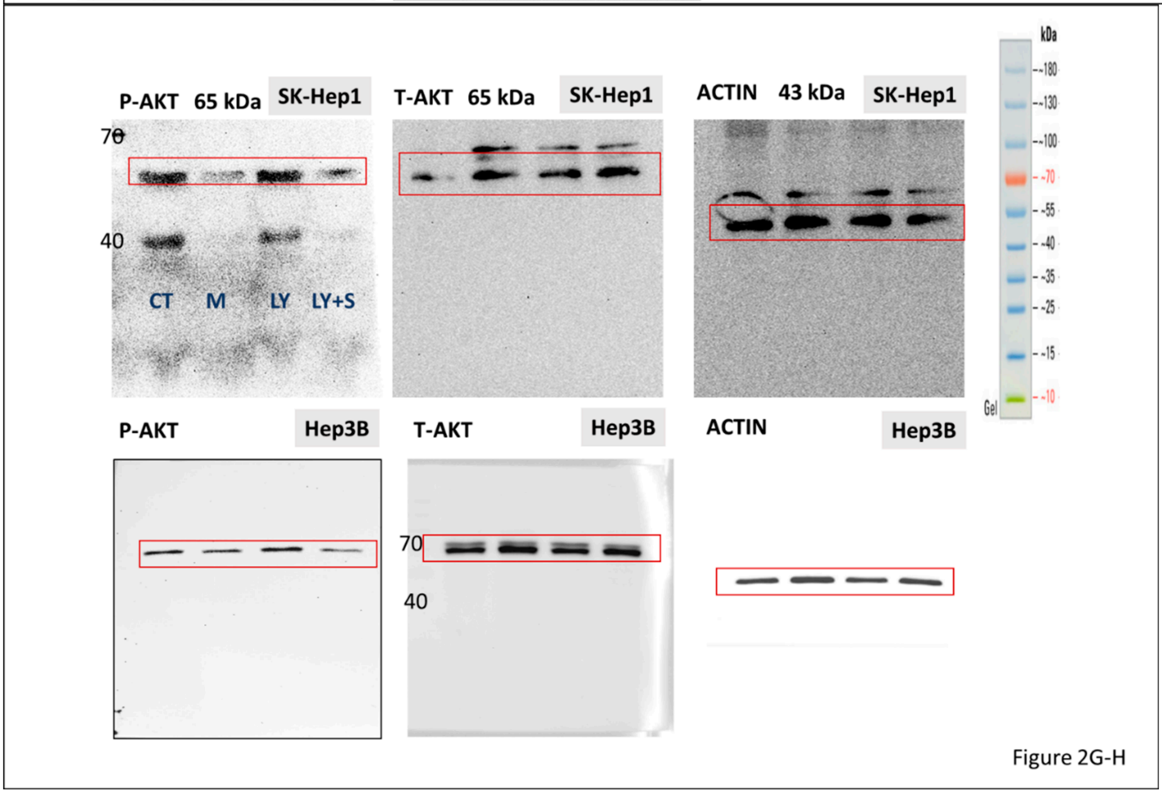

Figure 2G-H

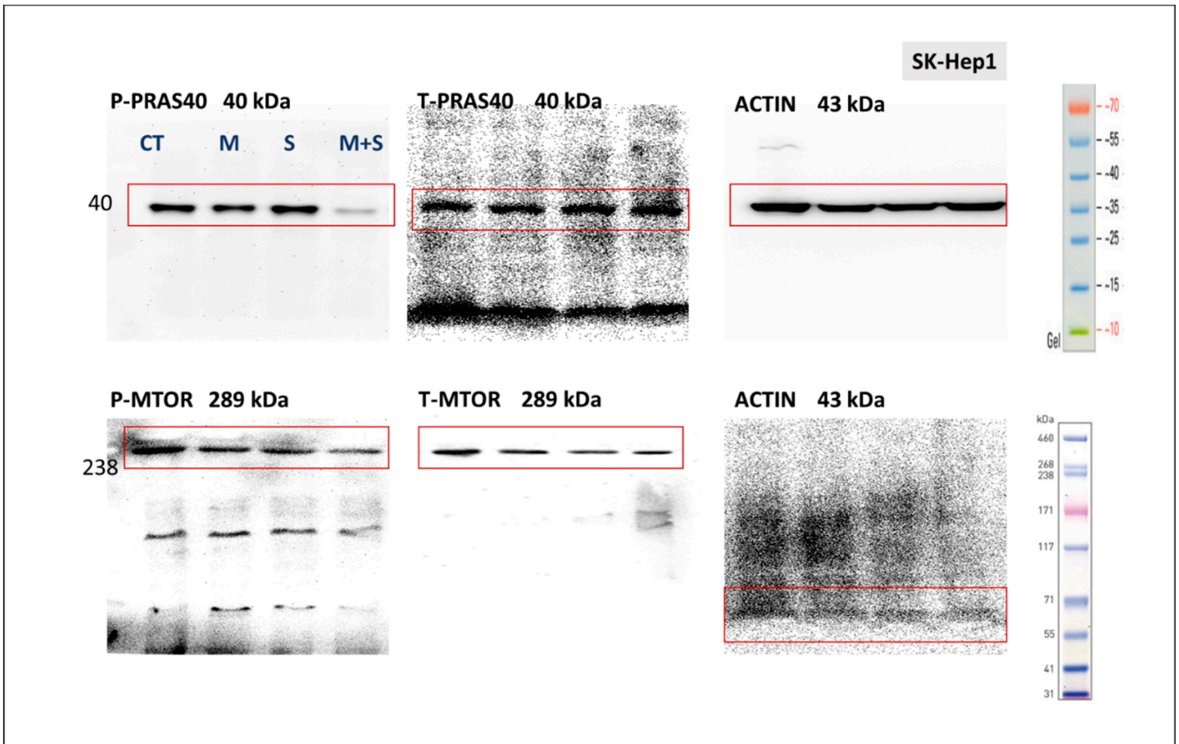

Figure 2I

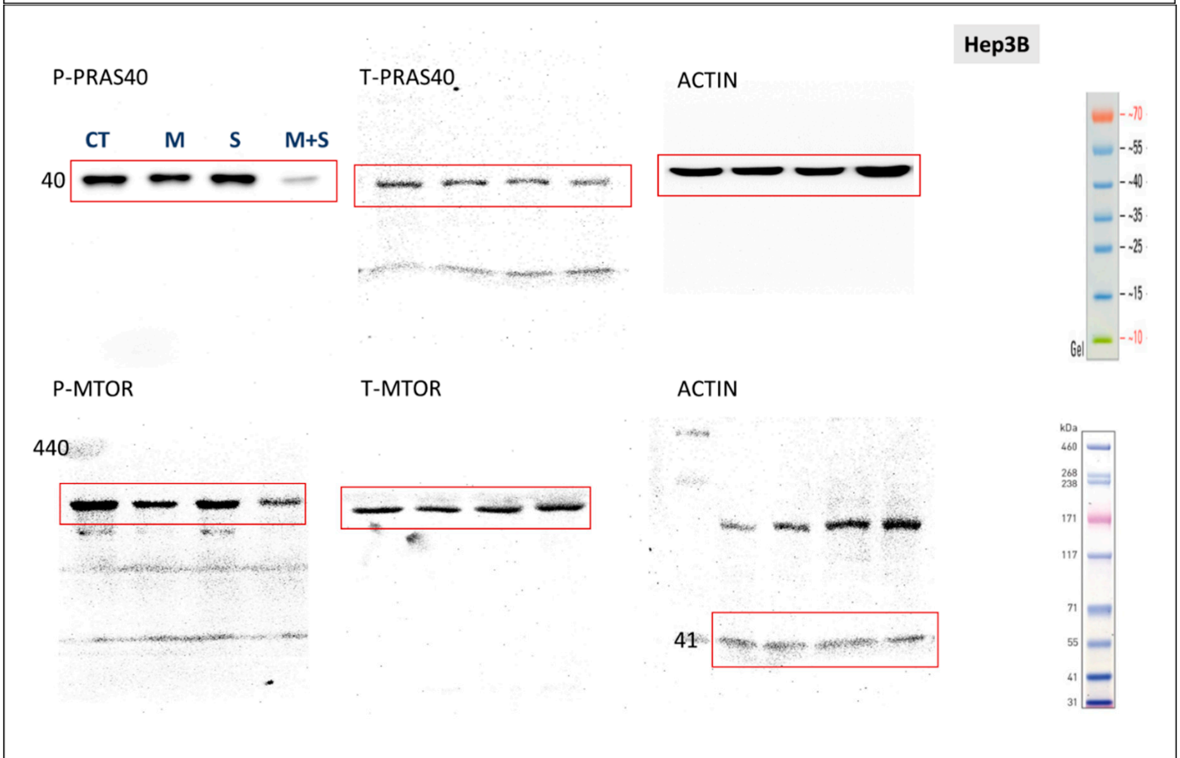

Figure 2I

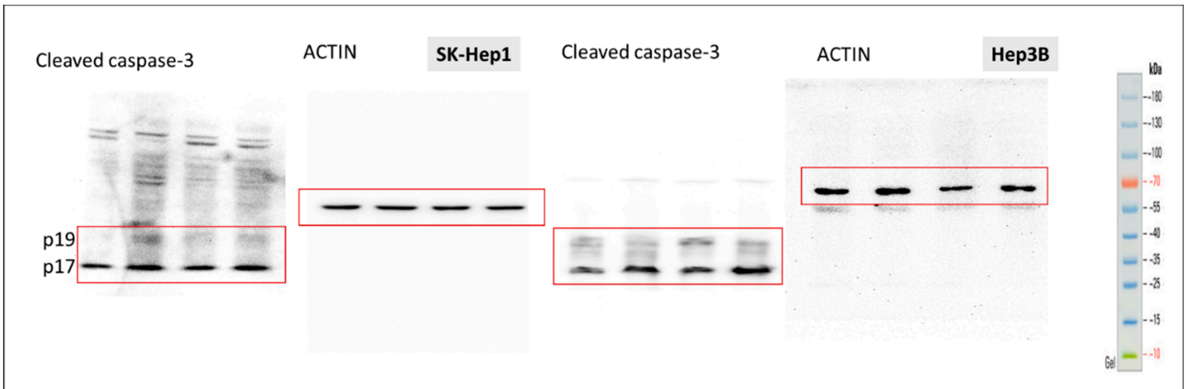

Figure 3G

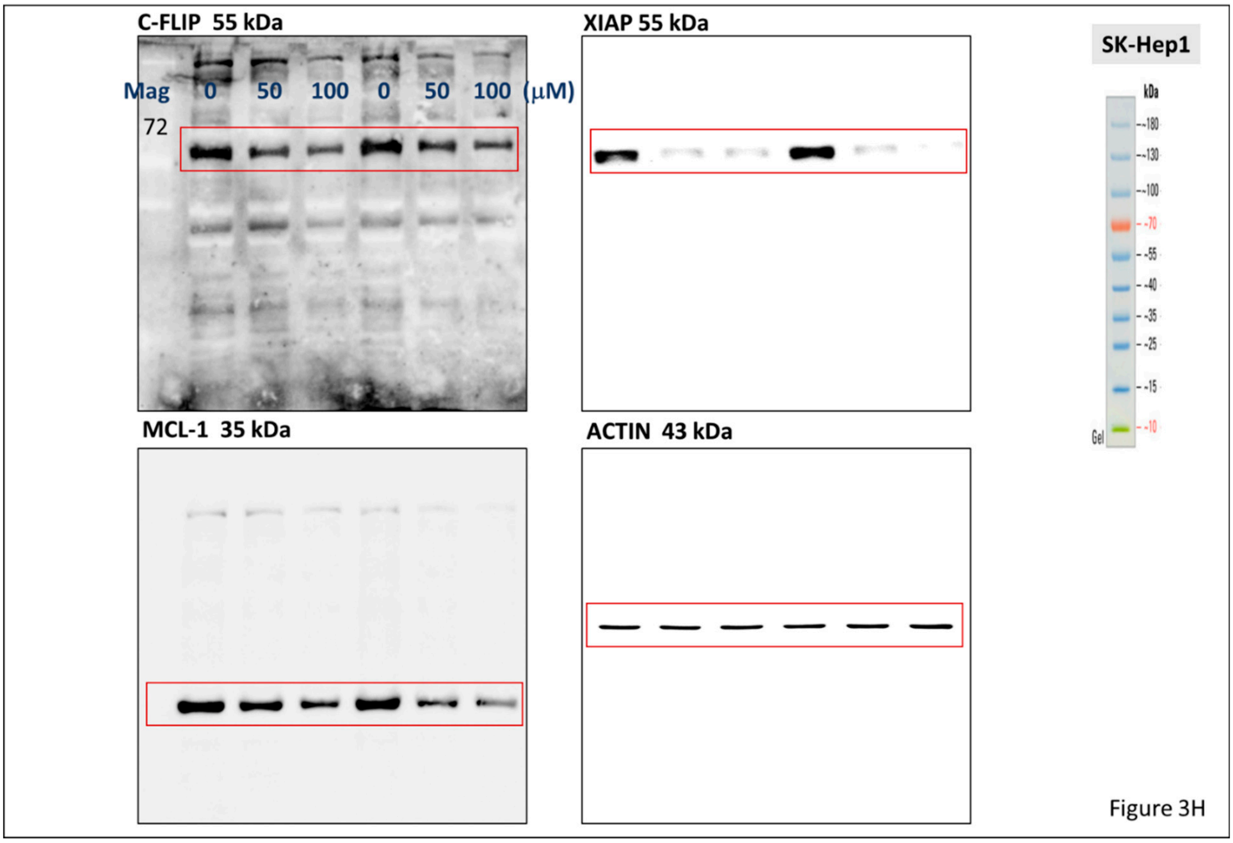

Figure 3H

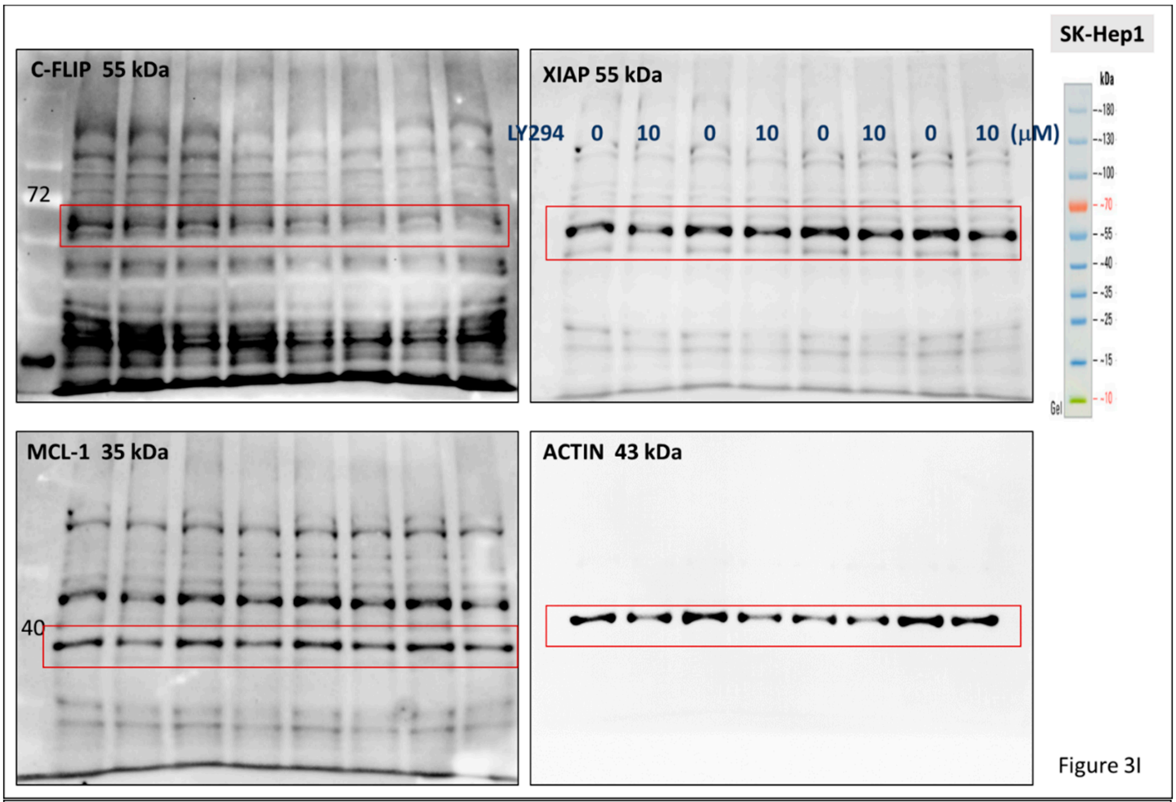

Figure 3I

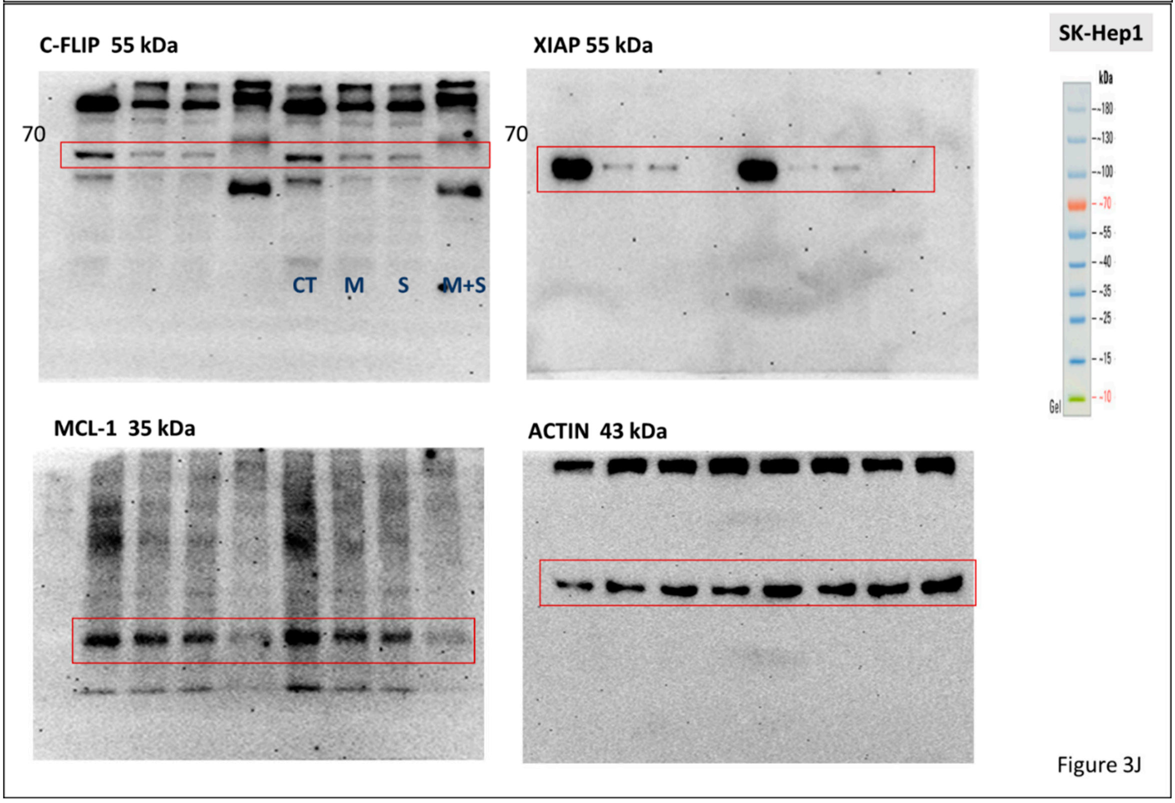

Figure 3J

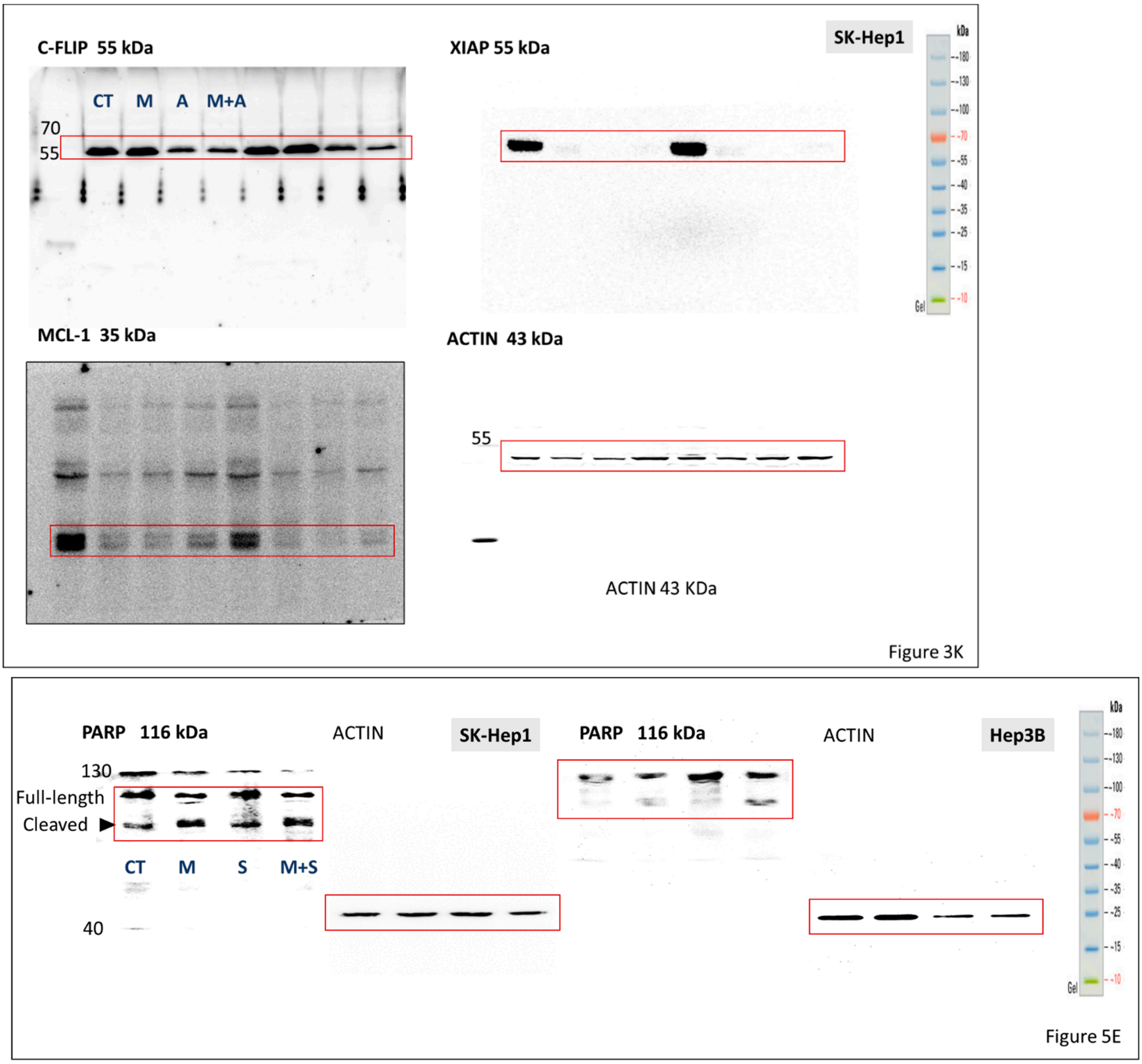

Figure 2. Full Western blot images.
